# Protein Conformational States—A First Principles Bayesian Method †

**DOI:** 10.3390/e22111242

**Published:** 2020-10-31

**Authors:** David M. Rogers

**Affiliations:** National Center for Computational Sciences, Oak Ridge National Laboratory, Oak Ridge, TN 37831, USA; rogersdm@ornl.gov

**Keywords:** Bernoulli mixture, Bayesian clustering, unsupervised classification

## Abstract

Automated identification of protein conformational states from simulation of an ensemble of structures is a hard problem because it requires teaching a computer to recognize shapes. We adapt the naïve Bayes classifier from the machine learning community for use on atom-to-atom pairwise contacts. The result is an unsupervised learning algorithm that samples a ‘distribution’ over potential classification schemes. We apply the classifier to a series of test structures and one real protein, showing that it identifies the conformational transition with >95% accuracy in most cases. A nontrivial feature of our adaptation is a new connection to information entropy that allows us to vary the level of structural detail without spoiling the categorization. This is confirmed by comparing results as the number of atoms and time-samples are varied over 1.5 orders of magnitude. Further, the method’s derivation from Bayesian analysis on the set of inter-atomic contacts makes it easy to understand and extend to more complex cases.

## 1. Introduction

The conventional description of protein dynamics asserts that proteins posses intrinsic conformational states [1]. An enzyme may cycle between catalytic and open states [2]. An ion channel may open and close its central pore [3]. A chaperone protein assists transformation of large, hydrophobic proteins from initial, linear, to folded shapes [4]. X-ray and cryo electron-microscopy reveals conformations with small motions on the 1–5 Ångstrom level for those proteins that crystallize [5]. Neutron scattering and nuclear magnetic resonance structures of room temperature proteins show greater shape variability, but are usually able to classify structures into a few ‘canonical’ structures.

Advances in molecular modeling have made it possible to simulate the protein folding process, generating very large numbers of samples, free energy landscapes, and information on kinetics. Nevertheless, computational identification of distinct conformational states from molecular simulations has remained an active area of methodological research. Approaches fall roughly into two classes—linear and nonlinear. Linear methods work with the real 3N-dimensional vector of atomic coordinates as a linear space, using pairwise alignments and Euclidean (RMSD) distances between structures. Nonlinear methods work with ‘features’ derived from nonlinear functions of the atomic coordinates—like pairwise distances between alpha-carbons. Well-known linear techniques include principle component analysis and linkage clustering based on pair-RMSDs. Nonlinear techniques include methods based on internal coordinates (relative distances and angles between groups of atoms) and T-distributed stochastic neighbor embedding [6].

Both linear and nonlinear techniques can be applied to subsets or groups of atoms to classify site-specific or inter-domain structures, respectively. This can be done trivially by handing off different point-sets to the analysis. Thus, most methods can be adapted to run with reasonable compute time and yield classifications most relevant to a motion under investigation. Automatically detecting protein secondary and tertiary structures, however, remains a challenging problem which we cannot address here.

One of the principle methods developed for visualizing domain motions in proteins is DynDom [7,8]. DynDom works from two input structures and determines relative domain rotations (using internal coordinates). This allows predicting transition motions from experimentally observed conformers, but not conformations from observed motions. On the other hand, many structure-to-structure similarity classification methods have emerged for this problem [9,10,11]. A recent review of such dimensionality reduction methods for protein conformational spaces noted that nonlinear methods are generally better than Cartesian or linear ones, but that the complexity of assumptions behind those models makes them difficult to work with and adapt [6,12].

This work presents a complete inference method derived from one single statistical hypothesis: that conformational states are defined by sets of contacting residues. Specifically, we hypothesize that the conformational state, *k*, uniquely determines which pairs of residues *u*, *v*, will be touching. Like a weighted coin flip, the contact probability is $\mu_{k,(u:v)}$—independently from all the other contacting pairs. Each conformational state is thus characterized by a vector, $\mu_{k}$, encoding the set of contacting pairs in state *k*.

The statistical model derived from this problem statement is termed a Bernoulli mixture model for binary feature classification [13]. The problem setup is similar to the Naive Bayes method [14]. However, because the categories are not known in advance, this is an unsupervised learning and classification problem.

Bernoulli mixture models have been applied extensively in the field of text subject analysis [15], optical character recognition [14], and image feature classification [13]. Essentially all of these applications have been successful at building extremely accurate classification models. The latter work also presents a thorough summary of sampling methods.

However, there remain difficulties sampling the distribution over categories, μ, especially when the number of categories and reference classifications are not known in advance. The well-known expectation-maximization algorithm (EM) [16] is available in principle, but is not a replacement for sampling. Theoretical work on the EM method [17] shows that redundant categories will result in many circumstances. In this work, we have introduced a prior that eliminates redundant categorizations.

This work is structured as follows. Section 2 presents the underlying probability distribution of categories, then outlines a novel method for quickly sampling parameter space—achieving category inference. Full technical details are present in Appendix A.1 and Appendix A.2. Section 3 describes test problems on which the method is demonstrated. Section 4 presents results demonstrating that the method creates structurally meaningful categories with >90% accuracy. Although the potential application space is vast, this work focuses on proving method robustness using well-defined synthetic test problems. Each follows a time sequence mimicking domain motions in proteins—so that the classification accuracy can be judged by correctly assigning categories in time-order. For the practitioner interested in trying the method directly, full source code and scripts reproducing the test cases in this work are available under the GPLv3 license (Supplementary Materials or [18]).

## 2. Theory

A naïve Bayes model (for bit-strings) assumes that structural input samples, i=1,…,N are generated by first selecting a conformational state, $z_{i}\in \left\{1,\ldots ,K\right\}$, with probability $\pi_{z_{i}}$, and then independently deciding whether each point-to-point contact ($x_{ij},j=1,\ldots ,M$), is made with probability $\mu_{z_{i}j}$. If contact *j* is made in sample number *i*, then $x_{ij}=1$. The bit-vector, $x_{i}$, is said to posess feature *j*. Otherwise $x_{ij}=0$, and the feature is absent. The model parameters are thus θ=(K,π,μ).

It leads to a sampling distribution,

(1)P(xz|θ)=∏iπzi∏j=1Fμzijxij(1−μzij)1−xij(2)=∏k=1KπkNk∏j=1MμkjNkj(1−μkj)Nk−Nkj.

The second line above notes that, once the categorization, *z*, is known, the sampling distribution is easy to express in terms of feature counts in $\left\{i:z_{i}=k\right\}$—the set of samples assigned to category *k*,

(3) $$\begin{array}{cccc} N_{k} & =|\{i:z_{i}=k\}|, & N_{kj} & =\sum_{\left\{i:z_{i}=k\right\}}x_{ij}. \end{array}$$

The first is the number of samples in set *k*, and the second is the number of times each contact is seen in that set.

According to Bayes’ theorem [19], we can turn this around to predict two important things—the probability that sample *i* belongs to category *k*, (read *z* given *x* and θ),

(4) $$P\left(z_{i}=k|x_{i}\theta \right)\propto \prod_{j=1}^{M}\mu_{kj}^{x_{ij}}{\left(1-\mu_{kj}\right)}^{1-x_{ij}},$$

and also the probability distribution over all possible parameters,

(5) P(θz|xI)=C(x)P(xz|θ)P(θ|I),

where C(x) is an *x*-dependent normalization constant. Sampling this distribution provides everything—the categorizations, *z*, the conformational states, π,μ, and even a predicted number of categories, *K*.

In Bayesian probability, a prior distribution has to be assumed by the researcher. The prior characterizes the parameter space, independently from any sampled data. Our prior distribution over parameters, introduced below, is P(θ|I). Since the parameters directly determine the sampling distribution, the prior does not affect it [P(xz|θ)=P(xz|θI)]. Note that this work juggles between two different priors, *I* and *U*, because the inference problem is simpler using P(θ|U), but P(θ|I) eliminates redundant solutions.

We choose a prior probability,

(6) $$\begin{array}{cc} P(\theta |I) & \propto P\left(\theta |U\right)\prod_{k<l}\left(1-B(\mu_{k},\mu_{l})\right), \end{array}$$

(7) $$\begin{array}{cc} P(\theta |U) & =\frac{\Gamma (K\alpha )}{\Gamma {\left(\alpha \right)}^{K}}\prod_{k=1}^{K}\pi_{k}^{\alpha -1} \end{array}$$

that enforces uniqueness of the categories. Here $B\left(\mu_{k},\mu_{l}\right)=B_{kl}$ is the Bhattacharyya similarity between distributions *p* and *q*,

(8) $$B\left(p,q\right)=\prod_{j=1}^{M}\sqrt{p_{j}q_{j}}+\sqrt{\left(1-p_{j}\right)\left(1-q_{j}\right)}.$$

If two categories share the same distribution, then p=q, and B(p,q)=1. This forces our estimator to return zero likelihood that $\mu_{k}=\mu_{l}$ for any k≠l.

The second part of Equation (7), P(θ|U), is a conventional prior used for Bernoulli mixture inference in the literature. We use α=M+1 throughout this work. Appendix A.1 contains a detailed justification for this choice. Essentially, it forces the likelihood for dividing a category into two parts to be asymptotically insensitive to the number of features, *M*. A proof of this fact, as well as a useful connection to the information entropy of compression is also present in Appendix A.1.

### Sampling Method

Our sampling method is traditional Markov Chain Monte Carlo using four types of moves: a recategorization move, where categories, *z*, are assigned according to P(z|θxI), a reclassification move, where θ is sampled from P(θ|zxU) and accepted with probability $\prod_{k<l}\left(1-B_{kl}\right)$, and one split and one join rule. The function, P(θ|zxU), referred to here is just P(θ|zxI) without the Bhattacharyya distance terms, and with a different constant prefactor.

For the split trial move, one of the categories, *k*, is split into two new categories. Every member of *k* is re-assigned into one of two new sets, labaled *L* or *R*. Join moves are the opposite of split moves. This re-categorization changes the set labels, *z*. Specifically, $z_{i}$ goes from *k* to *L* or *R* for every *i* formerly at $z_{i}=k$.

Generating split or join trial moves was done by randomly choosing either one category to split or choosing two categories to join. For splits, member *i* of category *k* is moved to set *L* with probability $\eta^{x_{ij}}{\left(1-\eta \right)}^{1-x_{ij}}$. We used η=0.9, but any η∈(0.5,1.0) should work in principle. If all elements end up in *L* or *R*, the partitioning is re-done. To concentrate splitting on productive cases, we did not attempt to split categories with $N_{kj}=0$ or $N_{kj}=N_{k}$. Immediately after splitting or joining categories, a reclassification move (re-assigning θ) was performed. Category split moves were accepted using the Metropolis criterion, which is the smaller of $P_{\mathrm{gen},\mathrm{join}}P_{\mathrm{split}}/P_{\mathrm{gen},\mathrm{split}}P_{\mathrm{joined}}$ or one. Explicit formulas for the move generation probabilities ($P_{\mathrm{gen},\mathrm{split}}$, etc.) are provided in Appendix A.2.

**Figure 1:**
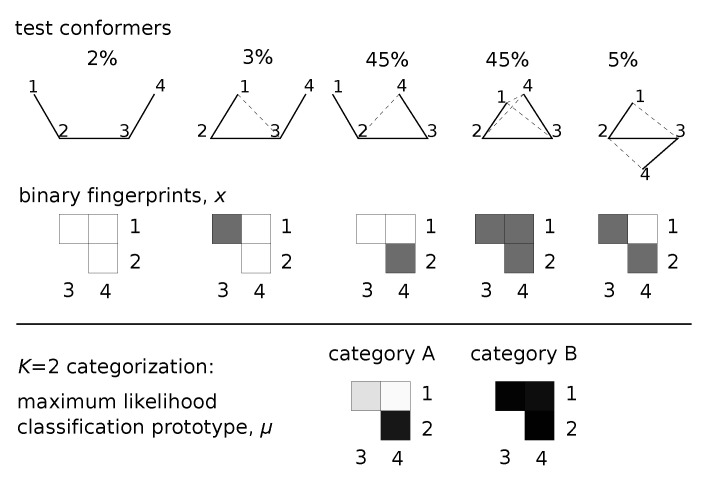
Classification applied to a 4-site structure. Five conformers (with frequencies shown) are input to the method. Bit-strings, *x*, for each conformer represent pairs of contacting residues (black squares correspond to dashed lines). Sampling the posterior distribution over (K,π,μ) provides a maximum-likelihood categorization at each *K*. We show both category prototypes, μ, determined by the algorithm for the K=2 classification. Darker shading in μ represents contacts with higher probability of forming.

Figure 1 provides a graphical summary of this inference scheme. Each conformational sample is mapped to a bit-string, which is used as the basis for inferring μ. Inference proceeds by sampling potential parameters until a good explanation for the data is found. Trial moves that re-categorize and update μ look horizontally to find better category prototypes. Trial moves that split categories based on presence or absence of some features allow us to traverse category space vertically.

## 3. Test Systems

The ability of our sampling procedure to predict categories was tested on three geometrical systems: ‘chomp’ (a closing angle), ‘heli’ (a rotating line), and ‘glob’ (three rotating spheres). Each system was generated as a time-series of 1000 frames for approximately *P* total particles in 2 or 3-dimensional space. After generation, Gaussian random noise of width σ=0.1 was added to every degree of freedom.

**Figure 2:**
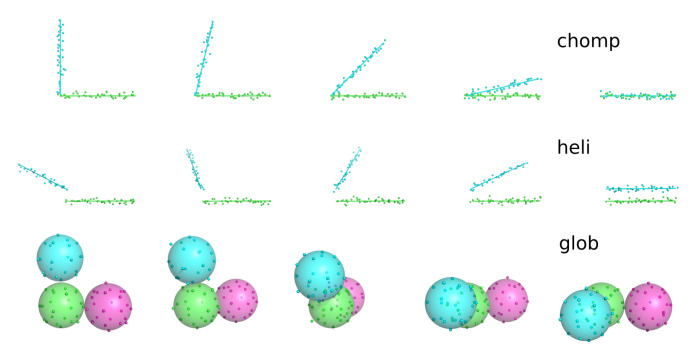
Sketch of the three synthetic motions used for testing. Lines, spheres, and colors are used to guide the eye, but the classification engine sees only the unlabeled points. The time axis proceeds left to right. For the ‘glob’ system, the sphere on the right moves behind, and then back to the right again while the top sphere moves slowly downward.

Figure 2 shows images of these three test trajectories. A complete description of the coordinate generation methods is present in Appendix A.3. All three systems were processed into binary feature data by calculating pairwise distances between all points. Pairs of points within 2 distance units were translated to 1 (representing contact). When forming the feature vectors, *x*, we removed features, *j*, for which every sample showed the same result (all contacting or all disconnected). It is important to note that this removal changes *M* seen by the algorithm—usually decreasing it well below P(P−1)/2.

Critically, we repeated these classifications for a range of material points, *P*. This tested robustness of the unsupervised classification problem with respect to the amount of features available. Adding more points without changing the geometry of the problem should not change the number of categories detected.

For each run, five independent MCMC chains were started from an assignment of all points to a single category. Each chain ran for 1000 steps. Samples were collected every 10 steps—starting from step 500. The acceptance probabilities for category split/join Monte Carlo moves varied around 10–13%.

## 4. Results

We analyzed the results of MC in two different ways. First, the categories assigned were tested for grouping in time. Since the contact lists (on which the categorization is based) varied slowly over time, we expect categories to come in ‘runs’. Second, we computed histograms over the number of categories, *K*. This is a strong test of the method’s sensitivity to the number of material points, *P*.

As expected, we found a high degree of correlation between categories and time for every case. Similar time-points were grouped into similar categories. To quantify these results, we counted transitions between category indices in time-order. For a perfect categorization, the number of transitions should equal the number of categories minus one. We computed the categorization accuracy in two ways. For each system, the left columns of Table 1 and Table 2 are 100 minus the percent of mis-categorized frames. For lossy categorization at time-boundaries, we expect oscillation between two values. We quantified this by forming a transition matrix between categories, and removing transitions along the ‘most likely path’. Excluding this boundary oscillation we found nearly 100% accuracy for the categorizations. Those are shown in the right columns of Table 1 and Table 2.

**Figure 3:**
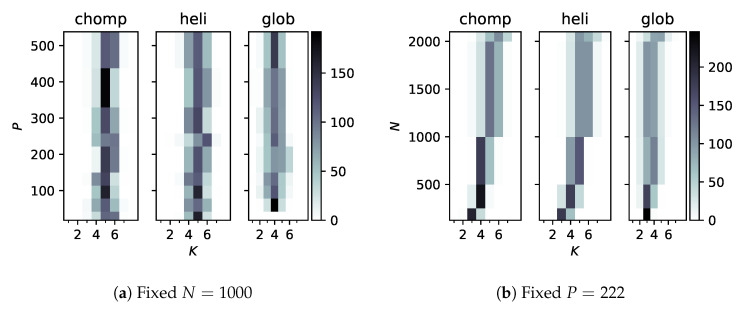
Counts (out of 250 samples each) of the number of categories, *K*, as *P* or *N* are varied. The shape of the distribution function remains essentially constant as *P* varies, but tends to spread toward higher *K* with more samples.

Integrating the posterior probability (Equation (2) times Equation (7)) over π leads to factors like $\Gamma {\left(N+\alpha K\right)}^{-1}$, which seem prohibitively costly as *N* increases. We therefore wanted to check that the number of categories does not decrease as features or samples are added. Figure 3 shows the sampled probability distributions over *K*, the number of categories, for increasing *P* (Figure 3a) and *N* (Figure 3b). Interestingly, for every system, about five conformational states were deduced at N=1000. As *N* increased, however, more categories were deduced and the distribution spread to higher numbers. This is probably reasonable, since more values of the ‘time’ coordinate generated a more fine-grained motion.

### Adenylate Kinase Open/Closed Transition

Finally, we tested the classification method against a protein with a well-characterized conformational transition. Adenylate kinase (ADK) converts ATP, ADP, and AMP by closing around substrate molecules [20]. The transition from closed to open was simulated in Reference [5] using steered molecular dynamics on a reaction coordinate interpolating between the electron density maps of PDB IDs 1AKE (Reference [20], closed) and 4AKE (Reference [2], open). The simulation data we used did not contain ligands, but did contain water and ions. Our analysis only made use of the alpha carbon (C${}_{\alpha }$) positions.

Features were calculated for each of N=3900 equally spaced frames during steered dynamics by testing whether C${}_{\alpha }$ to C${}_{\alpha }$ distances were less than 5 Å. These structures contain P=214 C${}_{\alpha }$-s. Sampling was carried out as described in Section 3, but 8 independent MC chains were sampled for 1250 steps (instead of 5 for 1000). The acceptance probability of split/join moves was 17%. During sampling, we saved the parameters, θ, possessing maximum likelihood values at each *K*.

Our implementation (Reference [18]) is parallelized so that each thread carries out an independent Monte Carlo chain. The run-time for the ADK example was less than 30 min. Larger runs on proteins up to 400 residues using 75,000 sampled protein conformations have been carried out using this method in under an hour. The implementation also contains a series of vizualization tools to highlight the protein regions responsible for detected conformation-to-conformation differences.

**Figure 4:**
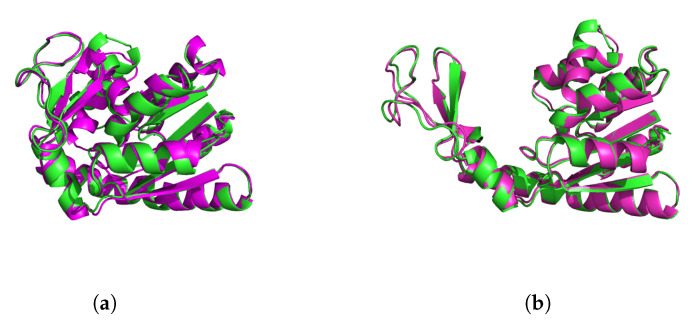
Adenylate kinase (ADK) closed (**a**) and open (**b**) configurations. PDB structures (magenta) are compared to the most similar molecular dynamics classification results (green) found among the five representative structures for the K=5 classification.

We then extracted conformational states with the highest probability for landing in each category as representative points for that category. Since the reference open and closed PDB conformations formed extreme points, our representative structures approached them more nearly as *K* increased. Figure 4 shows that the two end-point conformations ended up very close to the open and closed states from the PDB.

## 5. Conclusions

The method developed here is ideally suited for the unsupervised structural classification problem. It has been derived from a first-principles Bayesian analysis of the set of atoms which interact within a structure. This work solved the central problem of defining an appropriate prior distribution over parameter space and implementing an efficient sampling method.

Tests on sample conformational transitions identified more categories than naïvely expected because it generated milestones along the motion’s time-coordinate. However, all categorizations were shown to have excellent accuracy as judged by picking out the correct time-sequence. Finally, a test on Adenylate kinase verified that these conclusions easily generalize to protein motions.

The central results of this work showed that the method behaves well under large variations in the number of features and samples. These observations validate our choice for the prior distribution, since changes in P(θ|I) will have large effects on the distribution over number of categories, P(K|xI). They also show robustness of the MC sampling method itself, since relatively high acceptance rates were achieved.

Many future applications and the development of this method are possible. Changes in the pair contacts between states could be analyzed more thoroughly, as in the DynDom method [7]. Probabilities of assignment to each conformational state can be used as reaction coordinates. We are presently applying the method to classify chemical compound space using binary MACCS fingerprints [21], and to characterize conformational space of SARS-CoV-2 proteins simulated using replica exchange molecular dynamics [22].

Now that the principle has been demonstrated, more informative classification schemes can be devised. Adding information like hydrogen-bonding, salt bridge formation, and secondary structure annotation will allow the Bayesian framework able to recognize categories more like a biochemist would. The method can also be focused on active domains and binding sites by adding more points and shorter distance cutoffs for key residues. The insensitivity to *M* shown in this work provides a high degree of confidence that any amount of additional data will improve the overall categorization without spoiling the classification already achieved using coarser-level data.

## Figures and Tables

**Table 1:** Categorization accuracy for each system type and number of points, *P*. The left column for each system is the percent of category assignments identical to their previous time (not including K−1 required). The right column for each is the percent of category assignments that do not switch between runs.

*P*	Chomp		Heli		Glob	
18	89.2	98.0	89.9	98.9	91.1	96.7
42	94.7	98.9	93.7	99.0	93.5	97.3
78	96.3	99.3	95.3	99.4	95.5	98.0
114	96.8	99.4	96.5	99.5	96.4	98.4
150	97.2	99.4	96.7	99.4	97.0	98.5
222	97.8	99.5	97.0	99.7	98.2	99.1
258	98.4	99.7	97.7	99.5	98.6	99.4
330	98.4	99.6	97.8	99.7	98.7	99.4
438	98.5	99.7	98.1	99.5	98.8	99.4

**Table 2:** Categorization accuracy for each test system as the sample number, *N*, varies. The number of points remains fixed at P=222. Column labels are as in Table 1.

*N*	Chomp		Heli		Glob	
125	99.3	99.2	99.8	99.2	99.5	99.3
250	99.4	99.6	99.3	99.6	99.2	99.4
500	98.8	99.6	97.7	99.7	98.6	99.3
1000	97.7	99.6	96.9	99.6	98.2	99.2
2000	96.9	99.4	96.5	99.6	98.0	99.1

## Supplementary Materials

The source code for this classification method, along with the code to reproduce the test calculations in this paper, is publicly available from Reference [18].

## Appendix A.

### Appendix A.1. Behavior of the Posterior PDF with Increasing M

We argue here that the implicit uniform prior for all $\mu_{kj}$ as well as the choice α=M+1 is most appropriate for this problem. This is the conclusion we arrive at by requiring that the likelihood for dividing a category into two parts should be insensitive to *M*.

First, multiply P(xz|θ) and P(θ|U) from the main text to provide P(xzθ|U),

(A1) $$\frac{\Gamma (K\alpha )}{\Gamma {\left(\alpha \right)}^{K}}\prod_{k=1}^{K}{\left(\frac{\Gamma (2\beta )}{\Gamma {\left(\beta \right)}^{2}}\right)}^{M}\pi_{k}^{N_{k}+\alpha -1}\prod_{j=1}^{M}\mu_{kj}^{N_{kj}+\beta -1}{\left(1-\mu_{kj}\right)}^{N_{k}-N_{kj}+\beta -1}.$$

This is generalized a bit from the main text so that a Beta prior for μ (with parameter β) is explicitly shown.

Observe that (without the uniqueness constraint) the posterior distribution over categories is proportional to,

(A2) $$P\left(xz|U\right)\propto f\left(N,K\right)\prod_{k}\Gamma \left(N_{k}+\alpha \right)\prod_{j}\frac{\Gamma \left(N_{kj}+\beta \right)\Gamma \left(N_{k}-N_{kj}+\beta \right)}{\Gamma (N_{k}+2\beta )},$$

with $f\left(N,K\right)=\Gamma \left(\alpha K\right)/\Gamma {\left(\alpha \right)}^{K}\Gamma \left(N+\alpha K\right)$ (for β=1). The probability per category on the far-right can be likened to an information entropy,

(A3) Sinf(kj)≡lnNkNkj,

but is offset because of the limit,

(A4) $$\lim_{n\to \infty }\frac{\Gamma (n+a)}{\Gamma \left(n\right)n^{a}}=1.$$

Inserting this limit on the right, we find

(A5) $$\frac{\Gamma \left(N_{kj}+\beta \right)\Gamma \left(N_{k}-N_{kj}+\beta \right)}{\Gamma (N_{k}+2\beta )}\approx e^{-S_{\inf}\left(kj\right)}\frac{N_{kj}^{\beta -1}{\left(N_{k}-N_{kj}\right)}^{\beta -1}}{N_{k}^{2\beta -1}}.$$

This provides the motivation for choosing β=1. This choice for β makes the categorization closer to an information entropy, depending only on the ratio $N_{kj}/N_{k}$.

The categorization probability is now asymptotically like an information entropy, except for the extra factor of $1/(N_{k}+1)$. This factor is present for every feature, and creates an undesired dependence on *M*. We can cancel that factor by appealing to Equation (A4). This time using $n=N_{k}+1$ and a=α−1 to approximate $\Gamma (N_{k}+\alpha )$,

(A6) $$P\left(xz|U\right)\approx f\left(N,K\right)\prod_{k}\frac{N_{k}!{\left(N_{k}+1\right)}^{\alpha -1}}{{\left(N_{k}+1\right)}^{M}}\prod_{j}e^{-S_{\inf}\left(kj\right)}.$$

Equation (A6) provides the motivation for choosing α=M+1. With this choice, the ${\left(N_{k}+1\right)}^{M}$ term cancels. It leaves behind a relative information entropy for the $N_{k}$. Essentially,

(A7) $$P\left(xz|U\right)\approx f\left(N,K\right)N!\exp\left(S_{\inf}\left(\left\{N_{k}\right\}\right)-\sum_{kj}S_{\inf}\left(kj\right)\right).$$

At this point, different choices for f(N,K) lead to differences in the probability distribution over *K*, the number of categories. Rather than directly influencing these, we have chosen not to create additional assumptions about f(N,K) beyond the above choices for α and β.

### Appendix A.2. Sampling Method

Since π can be trivially integrated out of the posterior distribution (Equation (A1)), we re-generate π on every split/join move, and consider only the integrated probability P(zμ|xI) as our sampling target. Each time a split/join move is generated, a re-categorization of samples in category *k* ($\left\{x_{k}\right\}$) into $\left\{x_{L}\right\}$ and $\left\{x_{R}\right\}$. is immediately followed by generation of $\mu_{L}$ and $\mu_{R}$ (or $\mu_{k}$ for a join) from their respective Beta distributions (with samples $N_{Lj}$ and $N_{L}-N_{Lk}$, etc.).

We set up the acceptance probability for these split / join moves to satisfy the detailed balance condition,

(A8) $$\frac{P_{\mathrm{acc}}\left(z^{\prime }\mu^{\prime }|z\mu x\right)}{P_{\mathrm{acc}}\left(z\mu |z^{\prime }\mu^{\prime }x\right)}=\frac{P_{\mathrm{gen}}\left(z\mu |z^{\prime }\mu^{\prime }x\right)}{P_{\mathrm{gen}}\left(z^{\prime }\mu^{\prime }|z\mu x\right)}\frac{P(z^{\prime }\mu^{\prime }|xI)}{P(z\mu |xI)}.$$

Without loss of generality, assume $z^{\prime }$ contains K+1 categories and *z* contains *K*. We already have explicit formulas for P(zθ|xI) (modulo P(x|I)), so the only thing needed to calculate $P_{\mathrm{acc}}$ is the move generation probabilities.

Given category *k* and feature *j* was chosen to define the split (see main text for a description of this process), the recategorization probability is,

(A9) $$P_{\mathrm{gen}}\left(z^{\prime }|zkj\right)=\frac{1}{2}\left(\begin{array}{c} \eta^{N_{Lj}+N_{R}-N_{Rj}}{\left(1-\eta \right)}^{N_{Rj}+N_{L}-N_{Lj}}\\ \;+{\left(1-\eta \right)}^{N_{Lj}+N_{R}-N_{Rj}}\eta^{N_{Rj}+N_{L}-N_{Lj}} \end{array}\right)/\left(1-p_{L}-p_{R}\right).$$

The factor of $\frac{1}{2}$ is necessary because the labels *L* and *R* are interchangeable. $p_{L}$ ($p_{R}$) is the expression in the numerator when all $z^{\prime }$ move from *k* to *L* (R).

Because any *j* could have been chosen, the overall probability of ending up with categories *L* and *R* is,

(A10) $$P_{\mathrm{gen}}\left(z^{\prime }|z\right)=\frac{\xi (K)}{\sum_{nl}Q_{nl}}\sum_{j}Q_{kj}P_{\mathrm{gen}}\left(z^{\prime }|zkj\right).$$

To concentrate splitting on productive cases, we choose $Q_{kj}$ to be one for all k,j unless $N_{kj}=0$ or $N_{kj}=N_{k}$. In those cases $Q_{kj}$ is zero so that we do not split using a non-informative feature.

Generating $\mu_{L}$ and $\mu_{R}$ adds the factor,

(A11) $$P_{\mathrm{gen}}\left(z^{\prime }\mu^{\prime }|zx\right)=P_{\mathrm{gen}}\left(z^{\prime }|z\right)P\left(\left\{x_{L}\right\}|\mu_{L}\right){\left(N_{L}+1\right)}^{M}P\left(\left\{x_{R}\right\}|\mu_{L}\right){\left(N_{R}+1\right)}^{M}e^{\sum_{j}S_{\inf}\left(Lj\right)+S_{\inf}\left(Rj\right)}.$$

Here, we have made the intuitive definition,

(A12) $$P\left(\left\{x_{k}\right\}|\mu_{k}\right)\equiv \prod_{j}\mu_{kj}^{N_{kj}}{\left(1-\mu_{kj}\right)}^{N_{k}-N_{kj}}.$$

Most of this term will cancel P(zμ|xI) in the final expression for the acceptance probability.

The recombination rule is attempted with frequency 1−ξ(K+1). A set *u* is chosen at random with probability 1/(K+1) and recombined with set v≠u chosen with probability 2/K(K+1). The resulting generation probability is,

(A13) $$P_{\mathrm{gen}}\left(z|z^{\prime }x\right)=\frac{(1-\xi (K+1))2}{K(K+1)}.$$

Generating $\mu_{k}$ adds the factor,

(A14) $$P_{\mathrm{gen}}\left(z\mu |z^{\prime }x\right)=P_{\mathrm{gen}}\left(z|z^{\prime }x\right)P\left(\left\{x_{k}\right\}|\mu_{k}\right){\left(N_{k}+1\right)}^{M}e^{\sum_{j}S_{\inf}\left(kj\right)}.$$

As a final aid to computing the acceptance probability ratio (Equation (A8)), we provide a simplified expression for the likelihood ratio,

(A15) $$\begin{array}{cc} \frac{P(z^{\prime }\mu^{\prime }|xI)}{P(z\mu |xI)} & =\frac{\left(1-B_{LR}\right)\prod_{k^{\prime }\neq L,R}\left[\left(1-B_{Lk^{\prime }}\right)\left(1-B_{Rk^{\prime }}\right)\right]}{\prod_{k^{\prime }\neq k}\left(1-B_{kk^{\prime }}\right)}\frac{\Gamma (\alpha (K+1))}{\Gamma (\alpha K)\Gamma (\alpha )}\frac{\Gamma (N+\alpha K)}{\Gamma (N+\alpha (K+1))}\\ \; & \;\frac{\Gamma \left(N_{L}+\alpha \right)\Gamma \left(N_{R}+\alpha \right)}{\Gamma (N_{k}+\alpha )}\frac{P\left(\left\{x_{L}\right\}|\mu_{L}\right)P\left(\left\{x_{R}\right\}|\mu_{R}\right)}{P(\left\{x_{k}\right\}|\mu_{k})} \end{array}$$

### Appendix A.3. Coordinate Trajectory Generation for Synthetic Test Systems

The ‘chomp’ system was 2D, with two line segments, both of length 4 units. The bottom segment (P/2 uniformly spaced particles) stretched along the *x*-axis and remained stationary. The top segment (P/2−1 uniformly spaced particles, since the origin is excluded), moved at angle $\theta \left(t\right)=\frac{\pi }{4}\cos\left(t\right)$, t∈[0,π]. This motion mimicks a harmonic oscillator—which spends a majority of its time at the two limits of its motion.

The ‘heli’ system was 3D. It also had two line segments with P/2 equally spaced particles each. The bottom segment remained as before, while the top segment was replaced by P/2 particles displaced from the bottom one by 1 distance unit along *z*. The top segment was rotated in the xy plane with θ(t)=t, t∈[0,π].

The ‘glob’ system was 3D, consisting of 3 spheres, each represented by P/3 points at radius 2. The point locations were taken from Lebedev quadrature rules, which possess octahedral symmetry. Sphere 1 remained fixed at the origin. Spheres 2 and 3 started in locations displaced along *z* or *x*, respectively, by 2.1 distance units. As *t* traversed [0,π], sphere 2 was rotated about the origin around the *x*-axis by angle $\theta_{2}\left(t\right)=\frac{\pi }{4}\left(1-\cos\left(t\right)\right)$, while sphere 3 was rotated about the origin around the *z*-axis by angle $\theta_{3}\left(t\right)=\frac{\pi }{4}\left(1-\cos\left(2t\right)\right)$. This motion creates something like three conformational states. Since sphere 3 moves twice as fast as sphere 2, sphere 2 can be at +z or −y while sphere 3 is at +x. A third conformation is at the center where sphere 3 is at +y.

## References

[1] Guo J., Zhou H.-X. (2016). Protein allostery and conformational dynamics. Chem. Rev..

[2] Müller C.W., Schlauderer G.J., Reinstein J., Schulz G.E. (1996). Adenylate kinase motions during catalysis: An energetic counterweight balancing substrate binding. Structure.

[3] Amaral C., Carnevale V., Klein M.L., Treptow W. (2012). Exploring conformational states of the bacterial voltage-gated sodium channel NavAb via molecular dynamics simulations. Proc. Nat. Acad. Sci. USA.

[4] Kim Y.E., Hipp M.S., Bracher A., Hayer-Hartl M., Hartl F.U. (2013). Molecular chaperone functions in protein folding and proteostasis. Annu. Rev. Biochem..

[5] Vant J.W., Sarkar D., Fiorin G., Skeel R., Vermaas J.V., Singharoy A. (2020). Data-guided multi-map variables for ensemble refinement of molecular movies. bioRxiv.

[6] Spiwok V., Kříž P. (2020). Time-lagged t-distributed stochastic neighbor embedding (t-SNE) of molecular simulation trajectories. Front. Mol. Biosci..

[7] Lee R.A., Razaz M., Hayward S. (2003). The DynDom database of protein domain motions. Bioinformatics.

[8] Girdlestone C., Hayward S. (2016). The DynDom3D webserver for the analysis of domain movements in multimeric proteins. J. Comput. Biol..

[9] Stamati H., Clementi C., Kavraki L.E. (2010). Application of nonlinear dimensionality reduction to characterize the conformational landscape of small peptides. Proteins Struct. Funct. Bioinf..

[10] Ramanathan A., Savol A.J., Langmead C.J., Agarwal P.K., Chennubhotla C.S. (2011). Discovering conformational sub-states relevant to protein function. PLoS ONE.

[11] Ferguson A.L., Panagiotopoulos A.Z., Kevrekidis I.G., Debenedetti P.G. (2011). Nonlinear dimensionality reduction in molecular simulation: The diffusion map approach. Chem. Phys. Lett..

[12] Duan M., Fan J., Li M., Han L., Huo S. (2013). Evaluation of dimensionality-reduction methods from peptide folding–unfolding simulations. J. Chem. Theory Comput..

[13] Li C., Wang B., Pavlu V., Aslam J. Conditional Bernoulli mixtures for multi-label classification. Proceedings of the 33rd International Conference on Machine Learning.

[14] Alabau V., Andrés J., Casacuberta F., Civera J., Adrià Giménez J.-H., Juan A., Sanchis A., Vidal E. (2005). The Naive Bayes Model, Generalisations and Applications.

[15] Novovičová J., Antonín Malík A. (2004). Text document classification based on mixture models. Kybernetika.

[16] Kaji D., Watanabe K., Watanabe S. (2010). Phase transition of variational bayes learning in Bernoulli mixture. Aust. J. Intell. Inf. Proc. Syst..

[17] Yamazaki K., Kaji D. (2013). Comparing two Bayes methods based on the free energy functions in Bernoulli mixtures. Neur. Netw..

[18] Rogers D.M. Frobnitzem/Classifier: Classifier Version 1.0 (Version v1.0). Zenodo.

[19] Jaynes E.T. (2003). Probability Theory, The Logic of Science.

[20] Müller C.W., Schulz G.E. (1992). Structure of the complex between adenylate kinase from escherichia coli and the inhibitor ap5a refined at 1.9 å resolution. J. Mol. Biol..

[21] Durant J.L., Leland B.A., Henry D.R., Nourse J.G. (2002). Reoptimization of MDL keys for use in drug discovery. J. Chem. Inf. Comput. Sci..

[22] Acharya A., Agarwal R., Baker M., Baudry J., Bhowmik D., Boehm S., Byler K., Coates L., Chen S.Y., Cooper C.J. Supercomputer-Based Ensemble Docking Drug Discovery Pipeline with Application to Covid-19. https://chemrxiv.org/articles/preprint/Supercomputer-Based_Ensemble_Docking_Drug_Discovery_Pipeline_with_Application_to_Covid-19/12725465.

